# The tumor microenvironment and immune responses in prostate cancer patients

**DOI:** 10.1530/ERC-21-0149

**Published:** 2021-06-15

**Authors:** J T W Kwon, R J Bryant, E E Parkes

**Affiliations:** 1 Department of Oncology, University of Oxford, Oxford, UK; 2 Nuffield Department of Surgical Sciences, University of Oxford, Oxford, UK

**Keywords:** prostate, tumour microenvironment, immunotherapy, personalised medicine, molecular subgroup

## Abstract

The landscape of cancer treatment has been transformed over the past decade by the success of immune-targeting therapies. However, despite sipuleucel-T being the first-ever approved vaccine for cancer and the first immunotherapy licensed for prostate cancer in 2010, immunotherapy has since seen limited success in the treatment of prostate cancer. The tumour microenvironment of prostate cancer presents particular barriers for immunotherapy. Moreover, prostate cancer is distinguished by being one of only two solid tumours where increased T cell-infiltration correlates with a poorer, rather than improved, outlook. Here, we discuss the specific aspects of the prostate cancer microenvironment that converge to create a challenging microenvironment, including myeloid-derived immune cells and cancer-associated fibroblasts. By exploring the immune microenvironment of defined molecular subgroups of prostate cancer, we propose an immunogenomic subtyping approach to single-agent and combination immune-targeting strategies that could lead to improved outcomes in prostate cancer treatment.

## Introduction

Prostate cancer (PCa) is the second most commonly diagnosed cancer and the fifth most common cause of death in men worldwide (Sung *et al.* 2021). Although the aetiology of PCa is multifactorial, genomics is a key variable underpinning the development of this malignancy. Meta-analysis of genome-wide association data depicted an increase in incidence of PCa in men of African ancestry and the estimated lifetime risk of developing PCa is compounded by a positive familial history (Conti *et al.* 2021). Whilst many cases of low-risk volume PCa may be managed by active surveillance and not need immediate treatment, clinically significant localised and locally advanced PCa may be treated with curative intent by surgery or radiotherapy with concomitant androgen deprivation therapy (ADT). However, despite early-stage disease having a 5-year relative survival rate of approximately 100%, patient survival drops markedly to around 30% once PCa has disseminated (Siegel *et al.* 2020). Furthermore, advanced-stage disease in most cases develops resistance to ADT, underscoring the unmet clinical need to develop new treatments for metastatic castration-resistant PCa (mCRPC).

Although the effects of DNA repair defects and genetic/epigenetic aberrations on the cell division machinery are increasingly well-defined, it has become evident that the tumour microenvironment (TME) plays a pivotal role in the survival and propagation of tumours (Hinshaw & Shevde 2019). The TME comprises supporting stromal cells, endothelial cells and immune cells, each of which contributes to cancer development and behaviour. Lifestyle factors, such as microbiota and diet, which play key roles in the TME, have also been associated with prostate tumourigenicity (de Bono *et al.* 2020). By better characterising the TME, in particular the immune populations resident within these niches, we can begin to elucidate the immunosuppressive circuits that facilitate immune evasion by cancer cells. Crucially, identifying such resistance mechanisms creates an opportunity for the use of immunotherapies in effectively 'switching on' the immune system to recognise and eradicate cancers.

There are currently two immunotherapies approved by the US Food and Drug Administration (FDA) for PCa treatment. Sipuleucel-T is an autologous active cellular immunotherapy approved by the FDA in 2010 for the treatment of asymptomatic or minimally symptomatic mCRPC. This first-in-class cancer vaccine utilises patient-derived peripheral blood mononuclear cells (PBMCs) that have been stimulated *ex vivo* with a recombinant fusion protein consisting of prostatic acid phosphatase and granulocyte-macrophage colony-stimulating factor. Following leukapheresis and priming of antigen-presenting cells, the PBMCs are reinfused every 2 weeks for a total of three infusions. Results from the critical IMmunotherapy for Prostate AdenoCarcinoma Treatment (IMPACT) phase III study showed a significant improvement of 4.1 months in median overall survival (OS) in the treatment group compared to the placebo group. However, no significant difference was observed in median time to objective disease progression between the two patient cohorts (Kantoff *et al.* 2010).

The humanised monoclonal anti-PD-1 antibody, pembrolizumab, received accelerated approval from the FDA in 2017 for the treatment of unresectable or metastatic solid tumours. Of note, tumours needed to exhibit high microsatellite instability (MSI-H) or mismatch repair deficiency (dMMR). Supplementary to this molecular profile, requirements include disease progression following conventional therapy and a lack of viable alternative treatment options. Anti-PD-1 checkpoint blockade in the context of MSI-H/dMMR tumours is proposed to enhance prognosis by virtue of the hypermutated tumour phenotype possessing a greater neoantigen load and in turn, being associated with increased immune infiltration (Graham *et al.* 2020). Approval was predicated on the pooled analysis of patients enrolled across five clinical trials (KEYNOTE-012, -016, -028, -158 and –164) who were receiving pembrolizumab at either 10 mg/kg every 2 weeks or 200 mg every 3 weeks. Data from 149 patients yielded an objective response rate of 39.6% (95% CI: 31.7–47.9) with 78% of responses lasting 6 months or more (Marcus *et al.* 2019).

Both of these FDA-approved therapies have set important precedents in the immuno-oncology field. Sipuleucel-T was the first cancer vaccine and immunotherapy licensed for the treatment of PCa, while pembrolizumab was the first immunotherapy approved for a tumour-agnostic indication. Yet even with the advent of cancer vaccines and the success of immune checkpoint blockade (ICB) in other solid tumours such as melanoma, single-agent therapies have had limited success in the PCa setting (Kwon *et al.* 2014). This is thought to be due to the putative immunologically 'cold' nature of PCa, which tends to be characterised by restricted CD8^+^ T cell infiltration and low tumour mutational burden (TMB) (Alexandrov *et al.* 2013, Kaur *et al.* 2018).

Prostate tumours are also able to interact with supporting extracellular matrix and stromal elements to establish a chronic inflammatory and immunosuppressive milieu that is conducive to tumour growth. Herein, we review the PCa microenvironment and discuss immune subtypes typically present in PCa. Moreover, we propose a novel immunogenomic classification strategy to inform combination immunotherapy approaches for PCa.

## Immune cells in the prostate TME

### T cells

The inception of ICB has ushered in a new era of immuno-oncology where immune cells have replaced cancer cells as the principal focus of therapy. Of these immune cells, T cells, particularly cytotoxic T lymphocytes (CTLs), are most obviously vital during immune-mediated clearance of tumours (Van Allen *et al.* 2015). Tumour-infiltrating lymphocytes (TILs) are key mediators of anti-tumour immune responses and maintenance, with high TILs correlating with improved prognosis in breast cancer, colorectal cancer, melanoma and other solid organ malignancies (Mlecnik *et al.* 2011, Loi *et al.* 2019). This has led to the development of the 'Immunoscore', where immune infiltrate is quantified as a prognostic classification for solid tumours (Bruni *et al.* 2020). PCa, however, is distinguished by being one of only two tumour types (the other being renal cell carcinoma) where increased CD8^+^ T cell infiltration correlates with poor prognosis (Kaur *et al.* 2018, Petitprez *et al.* 2019). In these studies, PCa with increased CD8^+^ T cell density demonstrated earlier biochemical and metastatic relapse, and poorer OS. The reasons for this are currently unclear, with some data suggesting that highly T cell-infiltrated regions are associated with co-deletion of *BRCA2* and *RB1*, which are typically features of highly aggressive, poor prognosis PCa (Calagua *et al.* 2021). The specific contribution of T cell infiltration to this prognostic outcome requires further characterisation. Interestingly, T cell infiltrated regions of PCa lymph node metastases have dysfunctional signalling patterns, with high expression of exhaustion markers such as PD-1 and TIM-3 (He *et al.* 2021). Although PCa is often described as immunologically 'cold', a subset of immune-infiltrated cases exists, but with distinct T cell signalling patterns. Therefore, a personalised approach to considering immunotherapy is required. This distinct poor prognostic role of TILs in PCa, in contrast with most solid tumours, may start to explain the limited success of T cell targeting therapies in PCa potentially even in immune-infiltrated cases.

Despite a minority of PCa cases presenting with increased T cell infiltration, the typical intra-tumoural microenvironment of PCa presents with reduced T cell density, especially in the CD8^+^ subset. This exclusion of T cells from the TME assists in forming an immune-privileged site (Philippou *et al.* 2020, Wu *et al.* 2020), with immune exclusion a key mediator of resistance to ICB (Mariathasan *et al.* 2018). Hypoxic cores, commonly present in PCa, are also devoid of T cell infiltrate and provoke aberrant angiogenesis programmes that cultivate neovasculature capable of impeding CTL extravasation (Motz *et al.* 2014). Therefore, even upon gaining access to the tumour, T cells are typically confronted by a highly suppressive PCa TME (Shalapour *et al.* 2015, Bezzi *et al.* 2018, Jayaprakash *et al.* 2018).

The organ specificity of the microenvironment may additionally bear importance. The bone is the most common metastatic site in PCa, and it is often the case that bone metastases are far less responsive to ICB than metastases situated in soft tissues (Beer *et al.* 2017, Halabi *et al.* 2016, Sharma *et al.* 2019). Bone resorption during osseous disease results in the release of sequestered transforming growth factor beta (TGFB), which together with interleukin 6 (IL-6) produced by bone marrow stromal cells, can act on resident T cells to preclude tumour killing (Jiao *et al.* 2019).

### B cells

T cells are not the only immune population under consideration for mediating immunological treatment effectiveness. There is also a greater abundance of B cells in PCa compared to benign prostatic tissues, and the influx of B cells into the prostate TME has been linked with more aggressive disease (Woo *et al.* 2014, Wu *et al.* 2020). B cells, following their recruitment, can produce lymphotoxin, which activates IKKA-STAT3 and/or BMI1 signalling in residual cancer clones to hasten the onset of castration resistance and metastatic spread in PCa (Ammirante *et al.* 2010, 2013). In addition, IgA class-switched IL10^+^PD-L1^+^ plasma cells can attenuate CTL activation in a TGFB-dependent manner, thereby hindering anti-tumour activity (Shalapour *et al.* 2015). More recently, however, greater plasma cell content in primary prostate tumours has been associated with increased inflammation and prolonged recurrence-free survival in men of African ancestry, an outcome that has been related to increased IgG expression and NK cell activity (Weiner *et al.* 2021). These findings allude to how differential expression of cytokines in the prostate TME may influence the anti-tumour immune response and could explain the discrepancy in the role of B cells during PCa progression.

### Macrophages

A sizeable proportion of immune cells infiltrating PCa are from the myeloid lineage. Macrophages, traditionally dichotomised into either classical (M1) or alternative (M2) phenotypes, are one of the most prominent immune cell populations found within this malignancy. Their differing activation states can be achieved through various stimuli, IFNG or LPS in the case of the pro-inflammatory M1 phenotype and IL-4, -6, -13 or stromal cell-derived factor 1 (SDF-1) for the anti-inflammatory M2 phenotype (Comito *et al.* 2014, Di Mitri *et al.* 2019), but it is also possible for macrophages to be polarised through their effector functions, for instance efferocytosis promoting M2 polarity (Yang *et al.* 2015). Over-representation of M2 macrophages in prostatic tumours has been correlated with extracapsular extension and early biochemical relapse (Comito *et al.* 2014).

### Myeloid-derived suppressor cells

Other protagonists in the TME immune suppression phenotype of PCa are myeloid-derived suppressor cells (MDSCs), with it now apparent that MDSCs are able to govern tumour resistance to therapy. In *Pten*^PC*−*/*−*^, Myc-CaP and TRAMP-C1 allograft mouse models of PCa, MDSCs have been shown to be enriched in prostate tumours following surgical castration in a CXCR2-dependent manner. Of note, this intra-tumoural immune infiltrate was able to confer resistance in PCa cells to ADT by secreting IL-23, attesting to the potential of immunotherapies as a means of enhancing current endocrine treatments for CRPC (Calcinotto *et al.* 2018). Through co-stimulatory interactions with MDSCs, mast cells can further enhance immunosuppression via engagement of CD40 and directly impairing CD8^+^ T cell function (Jachetti *et al.* 2018).

MDSCs are a particularly attractive target for therapy, considering their pervasiveness in PCa and their profound effect on T cell functionality. The extent of T cell infiltration in prostate tumours has been described to have an inverse relationship with the frequency of MDSCs. Eliminating the MDSC population could maximise the T cell load available within PCa to promote anti-tumour responses (Lu *et al.* 2017). To this end, Lu* et al*. demonstrated using a chimeric mouse model of mCRPC that ICB is able to synergise with the tyrosine kinase inhibitors cabozantinib and BEZ235 to overcome *de novo* resistance. Tyrosine kinase inhibition was able to potentiate the effects of ICB by downregulating multiple cytokines that promote the expression of immunosuppressive genes in MDSCs and by selectively depleting this immune cohort from the TME.

### Neutrophils

Much like T helper (T_h_) cells and macrophages, neutrophils exhibit plasticity in their phenotype and can be polarised into anti-tumourigenic 'N1' or pro-tumourigenic 'N2' subtypes through various cytokines, including but not limited to G-CSF, IL-1B and TGFB (Fridlender *et al.* 2009, Casbon *et al.* 2015). Depletion of neutrophils in a mouse model of PCa metastasis augmented growth in bone, indicating a crucial role of this immune cell subset in promoting cancer growth. Furthermore, PCa appears to adjust to neutrophils over time with neutrophil cytotoxicity being gradually abrogated, correlating with prolonged neutrophil viability and diminished neutrophil extracellular trap formation (Costanzo-Garvey *et al.* 2020). These findings suggest that neutrophils may be able to combat the development of PCa metastases in bone, but caution must be exercised when devising therapies targeting these cells given the temporal dynamics of neutrophil effector function in the TME.

### Cancer-associated fibroblasts

Although not an immune cell, cancer-associated fibroblasts (CAFs) are major players in the immunosuppressive circuitry of tumours. Crosstalk of CAFs with M2 macrophages promotes prostate carcinogenesis and further still, M2 macrophages themselves stimulate CAF development by eliciting epithelial-to-mesenchymal transition in PCa cells and trigger neovascularisation (Comito *et al.* 2014). Additionally, ADT can induce hypoxia, which triggers autocrine TGFB signalling and trans-differentiation of CAFs into CXCL13-producing myofibroblasts (Ammirante *et al.* 2014). This subset of CAFs then recruits IgA^+^ plasmacytes that consequently impede CTL activity (Shalapour *et al.* 2015). The communication between cancer, immune and stromal cells presents a tight-knit community of resistance to immune-targeting treatments in PCa that requires further investigation.

## Androgen deprivation therapy and immune responses in prostate cancer

The androgen receptor is a crucial factor driving PCa progression, and anti-androgen therapies are a mainstay of treatment. ADTs reduce circulating testosterone by inhibiting gonadotrophin releasing hormone, inhibiting the androgen receptor or androgen synthesis via inhibition of CYP17 (Desai *et al.* 2021). The effect of both testosterone and androgen deprivation on the systemic and prostate immune responses is reviewed in (Gamat & McNeel 2017, Ben-Batalla *et al.* 2020) – ADT induces alterations in circulating T cells, increasing naïve and T_h_1 cells with a concomitant reduction in CD4^+^ T regulatory cells (T_regs_). Studies in mouse models indicate direct immunomodulatory effects of ADT on the PCa TME; however, depending on the model used (*Pten^−/−^* or MYC-driven, for example) distinct effects have been observed. A study of clinical samples of matched pre- and post-ADT early-stage PCa treated with neoadjuvant ADT indicated that treatment resulted in upregulation of immune checkpoints, such as PD-1 and CTLA-4, as well as infiltration of immune cells, including CD8^+^ and T_h_1 cells (Long *et al.* 2020). However, this relatively small study (six matched biopsies) was unable to delineate the effects of distinct PCa subgroups in early stage disease.

Combination ADT-immunotherapies are now in clinical trials for mCRPC, with promising results from early phase studies being investigated in large phase III studies, for example studying enzalutamide in combination with anti-PD-1 compared to enzalutamide with placebo (KEYNOTE-641 (Graff *et al.* 2021)). However, a study of enzalutamide in combination with anti-PD-L1 did not result in improved OS in mCRPC compared to enzalutamide alone (IMbassador250, Sweeney *et al.* 2020). Interestingly, a detailed single cell RNA-seq study using pre- and post-enzalutamide biopsies identified clonal expansion of T cells suggesting that response to enzalutamide (and tumour cell death) may be a pre-requisite for response to enzalutamide/anti-PD-L1/1 combination therapy (He *et al.* 2021). Additionally, although ADT is used across prostate adenocarcinomas, regardless of genomic subtype, it is not known if distinct subgroups of human PCa have differential immune responses to ADT. Moreover, the current role of ADT in combination immune therapy remains unclear, with further studies needed to determine the optimal combination of immune-targeting agents that synergise with ADT. The potential immunotherapeutic combination approaches in mCRPC is now an area of great clinical need, with approximately 41 ongoing clinical trials studying checkpoint inhibitor combinations in PCa (Bansal *et al.* 2021).

### Prostate cancer TME

**Figure 1:**
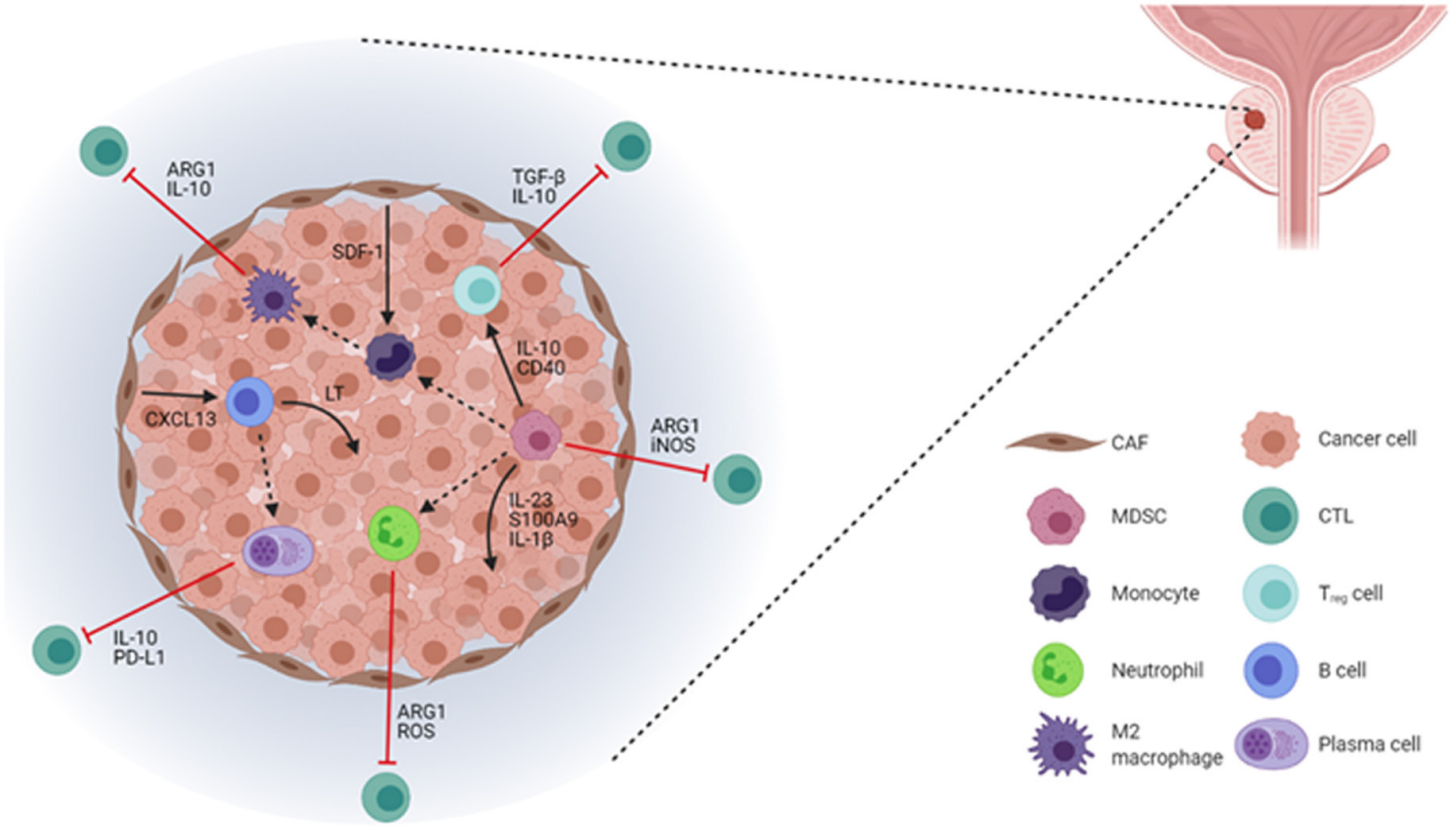
Immunosuppression and tumour-promoting circuitry within the prostate TME. Tumours are able to co-opt immune populations through various chemokine axes and initiate a complex interplay that facilitates tumour development. In addition to driving proliferation and survival of cancer cells through several soluble mediators, MDSCs upregulate suppressive factors that curb CTL activity while also being able to impede tumour killing through the recruitment of T_reg_ cells. Moreover, immature MDSCs can differentiate into either neutrophils or monocytes and subsequently M2 macrophages, all of which can further suppress CTL effector function. CAFs can additionally influence tumour immune contexture by recruiting B cells which not only support tumour growth and progression but also differentiate into immunosuppressive plasma cells. Alternatively, CAFs can instigate the differentiation of monocytes into M2 macrophages to sustain CTL inhibition. CAF, cancer-associated fibroblast; MDSC, myeloid-derived suppressor cell; CTL, cytotoxic T lymphocyte; LT, lymphotoxin; SDF-1, stromal cell-derived factor 1; ROS, reactive oxygen species; iNOS, inducible nitric oxide synthase; PD-L1, programmed death-ligand 1; S100A9, S100 calcium-binding protein A9.

When exploring the tapestry of the TME (Fig. 1 and Table 1), we need to consider the net effect of immune cells on carcinogenesis along with ways in which we can shift the balance of effector functionality in these resident populations to reinstate anti-tumour immunity. The complexity of the suppressive immune populations present in PCa and the role of the TME in promoting cancer growth indicate the multiple factors requiring consideration in developing novel immunotherapy approaches. The current immunotherapy trials in PCa have been reviewed extensively elsewhere (Fay & Graff 2020). However, the low tumour mutational burden of PCa and the limited success of current ICB strategies suggest a need for stratification of PCa for future therapies. Therefore, a combined immunogenomic classification may enable the selection of patients for tailored combination strategies.

**Table 1:** Immune cells in the prostate cancer microenvironment.

Cell type	Pro-tumourigenic activity
CAF	Trigger differentiation of monocytes into M2 macrophages (Comito* et al.* 2014); recruitment of B cells into TME (Ammirante* et al.* 2010, 2014)
T_reg_ cell	Inhibition of CTL effector function (Toso* et al.* 2014, Bezzi* et al.* 2018, Vidotto* et al.* 2019)
B cell	Promote growth and survival signalling in cancer cells (Ammirante* et al.* 2010, 2013); differentiation into plasma cells (Shalapour* et al.* 2015)
Plasma cell	Inhibition of CTL effector function (Shalapour* et al.* 2015)
MDSC	Promote growth and survival signalling in cancer cells (Bezzi* et al.* 2018, Calcinotto* et al.* 2018); recruitment of Treg cells into TME; differentiation into monocytes or neutrophils; inhibition of CTL effector function (Toso* et al.* 2014, Lu* et al.* 2017, Bezzi* et al.* 2018)
M2 macrophage	Inhibition of CTL effector function (Bezzi* et al.* 2018, Di Mitri* et al.* 2019)
Neutrophil	Inhibition of CTL effector function (Fridlender* et al.* 2009, Casbon* et al.* 2015, Costanzo-Garvey* et al.* 2020)

## Immunogenomic subtypes of prostate cancer

**Figure 2:**
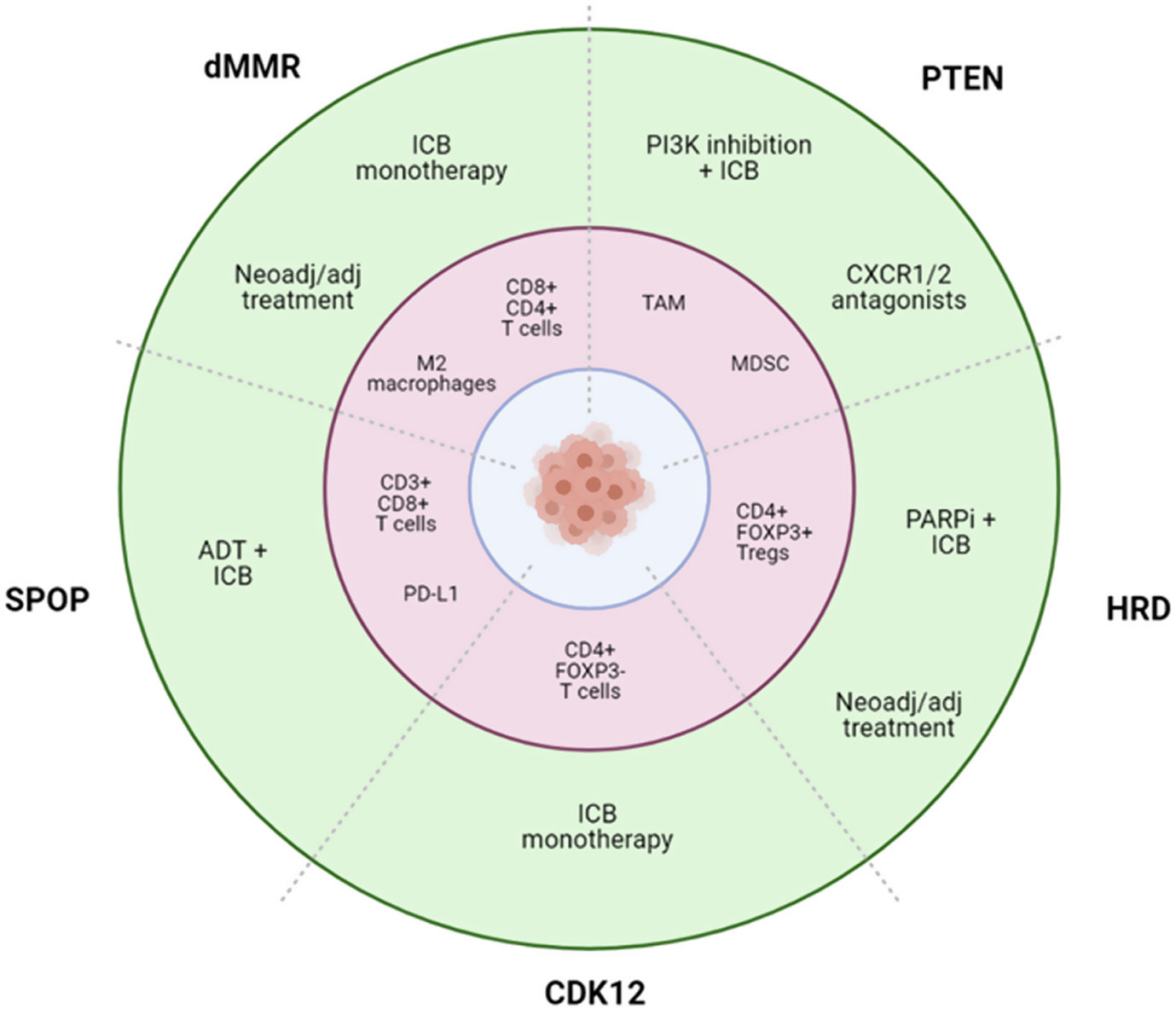
Immunogenomic subgroups and immunotherapeutic treatment strategies for PCa. Five immunogenomic subgroups of PCa are described. The inner ring (red) indicates the immune infiltrate characterised in each subgroup to date. Distinct immune populations are present in different genomic subtypes of PCa, indicating individual immune microenvironments to consider when designing immunotherapeutic treatment approaches. The outer ring (green) indicates potential treatment strategies for each subgroup. dMMR, microsatellite unstable/mismatch repair-deficient; PTEN, *PTEN*-deficient; HRD, homologous recombination-deficient; CDK12, *CDK12*-mutated; SPOP, *SPOP*-mutated; ADT, androgen deprivation therapy; ICB, immune checkpoint blockade.

As demonstrated in various solid tumours, the genomic characteristics of the tumour have a profound influence on the TME. While our proposed classification is not exhaustive, it provides a framework for considering personalised immunotherapy for PCa (Fig. 2).

## *PTEN*-deficient prostate cancer

Phosphatase and tensin homolog (*PTEN*) is one of the most commonly inactivated tumour suppressor genes in PCa, with around 20% of primary prostate tumours exhibiting aberrations in *PTEN*, rising to approximately 40% of mCRPC cases (Robinson *et al.* 2015). Somatic defects in *PTEN* are typically biallelic loss, with other inactivating point mutations, gene rearrangements and epigenetic changes occurring less frequently (Robinson *et al.* 2015). *PTEN* has a diametrically opposed role to phosphoinositide 3-kinase (PI3K), whereby it impedes signal transduction of the PI3K – Rac-alpha serine/threonine-protein kinase (AKT) – mechanistic target of rapamycin (mTOR) axis. As such, loss-of-function in *PTEN* in conjunction with amplifications and activating mutations in the PI3K pathway can synergise to promote higher-grade, more invasive PCa and tumour recurrence following treatment (Robinson *et al.* 2015). However, it is now becoming clear that the function of *PTEN* extends beyond its role in antagonising PI3K signalling to modulating inflammation and immunity (Li *et al.* 2016).

Several pre-clinical studies have revealed that deficient *PTEN* expression facilitates tumour development and resistance to therapy through the recruitment of MDSCs (Calcinotto *et al.* 2018, Zhao *et al.* 2020). Interestingly, concomitant deletion of certain genes altered the balance of immune cells subsisting within PCa. *Pten^PC^^−/−^*; *Zbtb7a^PC−/−^* tumours predominantly recruited polymorphonuclear-MDSC-like cells, which evoked tumour-promoting effects by stimulating NFKB signalling (Bezzi *et al.* 2018). *Pten^PC−^^/−^*; *Trp53^PC−/−^* tumours meanwhile recruited monocytic-MDSCs to enhance tumour growth by recruiting immunosuppressive T_regs_ and M2 macrophages. The latter mechanism of adaptive immune resistance was corroborated in another study where *PTEN* deficiency biased the PCa TME contexture towards greater FOXP3^+^T_reg_ numbers and overexpression of indoleamine 2,3-dioxygenase 1 (Vidotto *et al.* 2019). This chronic inflammatory and highly immunosuppressive state cultivated by MDSCs likely accounts for the dampened anti-tumour activity in the TME, even in the presence of cytotoxic effector T cells (Kaur *et al.* 2018). The same principles could be applied to the refractory nature of *PTEN*-deficient PCa to ICB, though an alternative explanation may be the ‘immune desert’ phenotype recapitulated, for example, by *Pten^PC^^−/−^; Pml^PC−^^/−^* tumours (Bezzi *et al.* 2018). Moreover, prostate-specific deletion of *Chd1* or *Erk5* on a *Pten^null^* background improved OS in mice relative to that solely lacking *Pten* expression; this prolongation in survival was attributed to curtailed chemoattraction of polymorphonuclear-MDSCs, thus permitting increased T cell infiltration into the TME (Zhao *et al.* 2017, 2020). As discussed above, therapies targeting MDSCs are promising in *PTEN*-deficient PCa, and are under clinical investigation in PCa more broadly.

Complete inactivation of *PTEN* is not only responsible for immunosuppression in the TME but also triggers senescence as a failsafe mechanism to counter prostate tumourigenesis (Chen *et al.* 2005). Several studies have reported that depletion of *PTEN* in PCa correlates with augmented expression of CXCL molecules, including CXCL1, CXCL2, CXCL5 and CXCL8 (Maxwell *et al.* 2013). The ensuing migration of monocytes/macrophages and myeloid cells via CXCR1/2-mediated chemotaxis into the TME subsequently promotes tumour progression, whether that be through initiating NFKB pro-survival signalling in tumour cells or by facilitating their escape from senescence (Armstrong *et al.* 2016). Pharmacological inhibition of CXCR2 was able to drive macrophages towards a pro-inflammatory state that induced growth arrest and senescence, specifically in prostate tumours deprived of *PTEN* expression (Di Mitri *et al.* 2019). These data demonstrate that blockade of CXCL-CXCR axes could serve as a viable treatment strategy for treating PCa patients that have been stratified according to *PTEN* status. However, caution should be exercised when leveraging senescence as a potential therapy approach. It has been evidenced that *PTEN*^null^ prostate tumours, through NFKB and JAK2/STAT3 signalling, can give rise to a senescence-associated secretory phenotype (SASP) geared towards the recruitment of MDSCs rather than lymphocytes (Toso *et al.* 2014). Indeed, Gr-1^+^ MDSCs have been shown to render the TME more permissive to tumour progression by antagonising the onset of senescence in prostate tumour cells (Di Mitri *et al.* 2014). PTEN-loss-induced senescence however does present opportunities for treatment – elimination of STAT3 signalling reconfigured the secretome of *Pten*^PC−^^/−^ senescent PCa to reactivate immune surveillance and heighten clearance of senescent tumour cells (Toso *et al.* 2014). Of note, abrogating the JAK2/STAT3 axis strengthened the pro-senescent effects of docetaxel on *PTEN*-deficient PCa and a similar synergy was observed when docetaxel treatment was combined with a CXCR2 antagonist (Di Mitri *et al.* 2014). Furthermore, ablating inhibitor of apoptosis protein (IAP) signalling sensitised *PTEN*^null^ prostate tumours to clinically relevant doses of ionising radiation (Armstrong *et al.* 2016). Such observations delineate the therapeutic utility of abolishing tumour cell-intrinsic pathways to bolster anti-tumour immunity and overcome chemo-/radioresistance afforded by *PTEN* deficiency in PCa.

Prostate tumours additionally partake in crosstalk with their encapsulating stroma in order to remodel the TME and achieve immune subversion. CXCL8 stemming from *PTEN* deficiency has been demonstrated to both augment autocrine CXCR4/7 and CCR2 expression in human PCa cells and instigate the paracrine release of their cognate ligands, CXCL12 and CCL2, by prostate stromal fibroblasts and monocytes (Maxwell *et al.* 2014). As well as upholding the survival and expansion of PCa, stromal-derived chemokines are likely involved in the recruitment and polarisation of other immune cell types that sustain tumour growth in the TME. A similar effect was observed in human PCa cells devoid of *PTEN* expression, where dysregulated AKT signalling enhanced CXCL12/CXCR4 expression by these cells and consequently amplified their proliferative and invasive capacities (Conley-LaComb *et al.* 2013). In models of bony metastases, stromal chemokines were able to induce AKT signalling in PCa cells and thus generate an osteolytic reaction *in vivo* that worsened tumour burden, perhaps by liberating immunomodulatory factors contained within the bone. Other studies demonstrated that loss of *PTEN* increased the expression of early growth response-1 (EGR1), a regulator of angiogenesis and osteoclast formation, across various PCa models (Li *et al.* 2019).

*PTEN* deficiency, therefore, has a role in modifying tumour niches to favour tumour development and this may, in part, explain the propensity that advanced *PTEN*-deficient prostate tumours have for the bone microenvironment. At the same time, loss of *PTEN* appears to relieve control on tumour progression and metastasis, giving a rationale to target the PI3K-AKT and chemokine axes when treating PCa. Combining PI3K-AKT targeting with immune checkpoint blockade, therefore, represents a potential immune-sensitising approach in this subgroup, with pre-clinical modelling of this strategy supporting the ability of PI3K inhibition to relieve *PTEN*-driven immunosuppressive activity and enhance immune responses (Qi *et al.* 2020).

## *SPOP* loss in prostate cancer

Speckle-type pox virus and zinc finger protein (*SPOP*) missense mutations resulting in functional loss of this tumour suppressor occurs in approximately 10–15% of PCa (Blattner *et al.* 2017). Similar to *PTEN* loss, *SPOP* mutations promote PI3K-mTOR signalling with subsequent enhanced cancer cell proliferation and growth. However, *SPOP-*mutated PCa appears to be a distinct genomic subgroup, co-occurring with *CHD1* deletions, and is associated with responsiveness to ADT, even in the metastatic setting (Swami *et al.* 2020). Interestingly, *SPOP* mutations result in increased PD-L1 levels in PCa, through the prevention of proteasome-mediated degradation (Zhang *et al.* 2018). This increased PD-L1 expression is closely associated with restriction of CD3^+^ and CD8^+^ T cell infiltration, promoting an immunosuppressive TME. Treatment of mouse models of *Spop-*mutant PCa with anti-PD-L1 resulted in responses, slowing tumour growth to the rate observed in wild-type *Spop* models.

The immune microenvironment of *SPOP*-mutant PCa, however, remains relatively uncharacterised in terms of myeloid lineage cells, which, as discussed above, are key mediators of immunoresistance in PCa. Additionally, the typically high levels of sensitivity to ADT in this subgroup mean that careful consideration is needed to determine the optimal combination immunotherapy approach. ADT itself modulates the TME, as discussed above, with upregulation of genetic modules associated with antigen presentation, immune checkpoint genes and IFN-γ signalling (Long *et al.* 2020). Similar to radiotherapy and chemotherapy, ADT is imprecise in terms of modulating immune responses, resulting in upregulation of both pro- and anti-tumourigenic immune pathways, which may indeed be PCa subtype-dependent. However, combination treatment with ADT and anti-PD-1 has shown promise in hormone-sensitive PCa, with single cell RNA-seq studies supporting the ability of this approach to optimise anti-tumourigenic immune responses (Hawley *et al.* 2020). Given the association of *SPOP*-mutant PCa with both ADT responsiveness and PD-L1 upregulation, this subgroup of PCa could be a promising target for this strategy.

## Mismatch repair-deficient prostate cancer

Around 3–12% of PCa can be classified as having dMMR with somatic and germline pathogenic alterations in MMR genes (Abida *et al.* 2019). Studies have suggested that dMMR PCa is associated with a higher Gleason score (79% Gleason 8–10) and metastatic disease (Graham *et al.* 2020). This is in contrast to colorectal cancer, where dMMR is associated with a good prognosis (Zaanan *et al.* 2018). This further highlights the unique factors of the PCa TME that need to be considered in optimising immunotherapy for this disease. Defects in the MMR pathway perturb the fidelity of the DNA replication process and give rise to genomic instability that mostly manifests within microsatellite regions. dMMR has therefore emerged as a predictive biomarker for ICB as the hypermutability associated with dMMR will increase the frequency of neoantigens capable of eliciting anti-tumour immunity. Studies of advanced PCa have shown that, of the dMMR mCRPC patients evaluable for objective responses, approximately 50% demonstrated durable biochemical responses with PSA reduction, and a similar proportion displayed no signs of radiographic progression following PD-1/PD-L1 blockade (Abida *et al.* 2019, Graham *et al.* 2020). Although responders tend to derive clinical benefit from continued treatment with ICB, some non-responders with dMMR status experienced deterioration in their condition post-treatment with anti-PD-1/PD-L1, suggesting there are other underlying mechanisms that govern response to ICB.

In keeping with dMMR in other solid tumours, most dMMR PCa demonstrate increased infiltration of CD8^+^ and CD4^+^ T cells, with increased expression of the immune checkpoint PD-L1, although notably a significant proportion of dMMR PCa (approximately 45% based on a small study of nine cases) do not demonstrate increased lymphocytic infiltration, potentially accounting for lack of response to ICB in some cases. In the same study, gene expression analysis suggested increased infiltration of M2-polarised macrophages (Nava Rodrigues *et al.* 2018). However, considering the overall positive response rates to ICB, and with pembrolizumab now FDA-approved for this subgroup of PCa, it is important to ensure timely testing of MSI status.

There is an argument for moving MSI testing to an earlier timepoint in the disease course – particularly given the success of neoadjuvant immunotherapy in the dMMR colorectal cancer setting (Chalabi *et al.* 2020). Immunotherapy in the neoadjuvant setting uses the tumour antigen load to encourage systemic long-lasting immune responses, targeting disease at a lower tumour burden. Taking this neoadjuvant approach in dMMR PCa is tantalising, offering a potential definitive first-line treatment for otherwise high-risk disease, although given the relatively low frequency of this subtype this would require an extensive testing strategy, and potentially optimising liquid biopsy companion diagnostic testing. A clinical trial investigating this approach could also explore potential biomarkers to identify patients who may benefit most from this strategy. A window study including combination (anti-CTLA-4 and anti-PD-1) or single-agent anti-PD-1 therapy could address the optimal approach for this subgroup. A platform study on this basis could also identify optimal immunotherapy combinations for dMMR PCa.

## Homologous recombination-deficient prostate cancer

Deficiency of homologous recombination DNA repair, required for faithful repair of double-strand DNA breaks arising as a result of stalled DNA forks or crosslinked DNA, occurs in approximately 5–8% of early-stage PCa, rising to 20–25% in the metastatic setting (Mateo *et al.* 2020). Similar to homologous recombination-deficient (HRD) ovarian and pancreatic cancers, PCa with mutations in key HR genes (including *BRCA1/2, PALB2, CHEK1/2,* and* ATM*) demonstrate sensitivity to poly(ADP-ribose) polymerase (PARP) inhibitors. In a landmark study, 711 patients were selected for molecular screening with 92 patients with metastatic PCa ultimately receiving the PARP inhibitor olaparib and being evaluable for response (Mateo *et al.* 2020). The highest response rates were observed in *BRCA1/2*-mutant PCa, and overall 57% of patients receiving the higher olaparib dose (400 mg) demonstrated clinical responses.

Treatment with PARP inhibition results in upregulation of interferon signalling, via activation of the STimulator of INterferon Genes (STING) pathway and promoting immune cell infiltration, and also increased immune checkpoint (PD-L1) expression (Vikas *et al.* 2020). Thus, the combination of PARP inhibition and ICB is a logical approach in HRD PCa, with a number of trials of this combination ongoing in mCRPC, with some evidence that this approach may even be effective in HR-proficient PCa (Karzai *et al.* 2018).

Even in the early stages, HRD PCa is an aggressive subtype, associated with a poor outcome following radical radiotherapy and moderate resistance to docetaxel (Taylor *et al.* 2017). *BRCA2-*mutant PCa, in particular, is associated with younger age at diagnosis of PCa, along with increased rates of both nodal and distant metastasis and a poorer prognosis. As above, it has been suggested that HRD PCa may be associated with increased lymphocytic infiltration, characterised by a CD4^+^ FOXP3^+^ T_regs_ intratumoural infiltrate and an increased CD8^+^ T cell infiltrate (Jenzer *et al.* 2019). A non-significant increase in CD163^+^ macrophages was also noted. This highly infiltrated immune TME alongside a dismal outlook for this subgroup of PCa justifies consideration of immune-targeting therapies at an earlier stage. Moreover, detailed characterisation of the TME in HRD PCa is required to select appropriate immune-targeting therapies. Given the low frequency of HRD in early stage PCa, a consortium approach could identify these tumours at an early stage, thereby permitting an appropriately powered study of the single cell immune landscape of HRD PCa and identification of appropriate immune strategies. A further limitation in this field is the lack of HRD *in vivo* models for PCa, and better pre-clinical models would enable in-depth immune analysis of this subgroup.

## *CDK12*-mutated prostate cancer

Although implicated in homologous recombination, cyclin-dependent kinase 12 (*CDK12*)-mutated PCa is genomically and immunologically distinct from HRD PCa, with limited PARP inhibitor responsiveness, high levels of genomic rearrangements, high neoantigen burden (second only to dMMR PCa) and increased lymphocytic infiltration (Antonarakis *et al.* 2020, Rescigno *et al.* 2021). In a large study of mCRPC, 4.7% were found to harbour *CDK12* alterations, associated with poorer OS. Characterisation of the TME by transcriptomic and immunohistochemical analysis demonstrating increased CD3^+^ T cell infiltration, with CD4^+^ FOXP3^-^ immunosuppressive T cells being the dominant population (Rescigno *et al.* 2021). Consistent with this observation, a small study of 9 patients receiving anti-PD-1 ICB demonstrated a 33% response rate, with 56% of patients remaining on treatment beyond 6 months (Antonarakis *et al.* 2020). These findings justify further characterisation of the myeloid compartment of these tumours, as well as investigation of *CDK12* alterations in forthcoming studies of ICB in PCa as a biomarker for stratification for single-agent ICB treatment.

## Conclusions

It is clear that PCa comprises more than one entity, with a number of immunogenomic subsets that could be exploited for therapeutic benefit using immunotherapy. A 'one-size-fits-all' approach is unlikely to result in successful clinical regimens, and consideration of the best approaches for detecting these subgroups is now needed in order to enable stratification for immunotherapeutic treatment. To that end, mining of clinical, genomic, transcriptomic and available histopathological data from existing trials of immunotherapy in PCa could enable determination of the appropriate subgroups for either single-agent ICB or specific combination strategies.

## Future directions

The discussions above demonstrate the principle of offering an immune-targeting treatment at the earliest possible stage of disease. The combination of heavily pre-treated disease, increased heterogeneity and established immunosuppressive networks in advanced metastatic PCa makes this a challenging population for immune-targeting treatments. Potential future clinical studies could focus on neoadjuvant window studies, using surrogate markers of response such as immune infiltration and detailed characterisation of immune responses, or *de novo* metastatic disease with lower tumour burdens. Early testing of tumours to detect, in particular, MSI or HRD could enable patients with these subgroups of disease to receive more aggressive immune-targeted treatment at an earlier stage, increasing the probability of long-term control and potential cure. The immunogenomic subgroups we propose above could be further stratified according to early or late-stage disease, and umbrella clinical trial designs incorporating ongoing biomarker discovery and validation used to select the appropriate immune combination treatments for each subgroup. This personalised immunotherapy approach could radically transform the future clinical outlook for men with PCa.

## Declaration of interest

The authors declare that there is no conflict of interest that could be perceived as prejudicing the impartiality of this review.

## Funding

J T W K, R J B and E E P are supported by funding from Prostate Cancer UK http://dx.doi.org/10.13039/501100000771 (Major Award in Immunology). R J B is supported by a Cancer Research UK http://dx.doi.org/10.13039/501100000289/Royal College of Surgeons of England http://dx.doi.org/10.13039/501100000297 Clinician Scientist Fellowship (reference C39297/A22748). E E P is supported by a personal fellowship (Young Investigator Award) from the Prostate Cancer Foundation http://dx.doi.org/10.13039/100000892 (2019).

## Author contribution statement

J T W K and E E P conceptualised the framework for this review. All authors contributed to writing and editing.
